# Naoxintong Protects Primary Neurons from Oxygen-Glucose Deprivation/Reoxygenation Induced Injury through PI3K-Akt Signaling Pathway

**DOI:** 10.1155/2016/5815946

**Published:** 2016-02-02

**Authors:** Yan Ma, Pei Zhao, Jinqiang Zhu, Chen Yan, Lin Li, Han Zhang, Meng Zhang, Xiumei Gao, Xiang Fan

**Affiliations:** ^1^Tianjin State Key Laboratory of Modern Chinese Medicine, Tianjin University of Traditional Chinese Medicine, Tianjin 300193, China; ^2^Institute of Traditional Chinese Medicine, Tianjin University of Traditional Chinese Medicine, Tianjin 300193, China; ^3^Second Affiliated Hospital of Tianjin University of Traditional Chinese Medicine, Tianjin 300150, China; ^4^School of Chinese Materia Medica, Tianjin University of Traditional Chinese Medicine, Tianjin 300193, China

## Abstract

Naoxintong capsule (NXT), developed from Buyang Huanwu Decoction, has shown the neuroprotective effects in cerebrovascular diseases, but the neuroprotection mechanisms of NXT on ischemia/reperfusion injured neurons have not yet been well known. In this study, we established the oxygen-glucose deprivation/reoxygenation (OGD/R) induced neurons injury model and treat the neurons with cerebrospinal fluid containing NXT (BNC) to investigate the effects of NXT on OGD/R induced neurons injury and potential mechanisms. BNC improved neuron viability and decreased apoptotic rate induced by OGD/R. BNC attenuated OGD/R induced cytosolic and mitochondrial Ca^2+^ overload, ROS generation, intracellular NO levels and nNOS mRNA increase, and cytochrome-c release when compared with OGD/R group. BNC significantly inhibited both mPTP opening and ΔΨ*m* depolarization. BNC increased Bcl-2 expression and decreased Bax expression, upregulated the Bcl-2/Bax ratio, downregulated caspase-3 mRNA and caspase-9 mRNA expression, and decreased cleaved caspase-3 expression and caspase-3 activity. BNC increased phosphorylation of Akt following OGD/R, while LY294002 attenuated BNC induced increase of phosphorylated Akt expression. Our study demonstrated that NXT protected primary neurons from OGD/R induced injury by inhibiting calcium overload and ROS generation, protecting mitochondria, and inhibiting mitochondrial apoptotic pathway which was mediated partially by PI3K-Akt signaling pathway activation.

## 1. Introduction

Stroke is the second cause of death and the leading cause of long-term disability in the world. Over the past 30 years, a huge amount of money has been spent in research and development of stroke therapeutics. The pathogenesis of ischemia/reperfusion injury is complex; calcium overload, oxidative/nitrosative stress, and mitochondrial dysfunction are involved as the main mechanisms of ischemia/reperfusion induced injury [1]. Although our knowledge is greatly enriched in understanding of the mechanisms of brain injury, repair, plasticity, and recovery after stroke, there is a remaining translational barrier between research benches and clinic beds. Intravenous tPA thrombolytic therapy is still the only FDA approved emergency treatment for acute ischemic stroke, when given within 4.5 h after stroke [2]. However, less than 5% of patients with ischemic stroke in the USA (2.4% in China) receive this treatment [3]. It is no doubt that developing neuroprotective medicine is clinically and extremely significant, especially to the treatment at late phase after stroke.

Meanwhile, traditional Chinese medicine, which was driven and developed mainly by clinical practice and proven efficacy, has the potential new treatments for stroke. Currently, there are more than 100 traditional Chinese patent drugs approved by Chinese National Drug Administration used clinically in China for stroke treatment and prevention [4]. Naoxintong capsule (NXT), developed from Buyang Huanwu Decoction [5, 6], is a traditional Chinese medicine approved by the Chinese National Drug Administration and used to treat patients with stroke and cardiovascular diseases [7–9]. NXT contains the following 16 various kinds of traditional Chinese medicines: Radix Astragali membranaceus (root of* Astragalus membranaceus* var.* mongholicus* (Bunge) Hsiao), Radix Paeoniae Rubra (root of* Paeonia lactiflora* Pall), Red sage (root of* Salvia miltiorrhiza*, Bunge), Radix Angelicae Sinensis (root of* Angelica sinensis* (Oliv.) Diels), Rhizoma Chuanxiong (root of* Ligusticum chuanxiong* Hort.), Semen Persicae (seed of* Prunus persica* Batsch), Flos Carthami (flower of* Carthamus tinctorius* L.), Frankincense (resin of* Boswellia carterii* Birdw or* Boswellia bhaw-dajiana* Birdw), myrrh (*Commiphora myrrha* (Nees) Engl. or* Commiphora molmol* Engl.),* Spatholobus suberectus* (vine stem of* Spatholobus suberectus* Dunn), Achyranthes Root (root of* Achyranthes bidentata* Blume), Cassia Twig (twig of* Cinnamomum cassia* J.Presl), Mulberry Twig (twig of* Morus alba* L.), Earthworms (*Pheretima aspergillum* (E. Perrier)), Scorpions (*Buthus martensii* Karsch), and Hirudo (*Whitmania pigra* Whitman), which were mixed at a ratio of 66 : 27 : 27 : 27 : 27 : 27 : 13 : 13 : 13 : 20 : 27 : 20 : 27 : 27 : 13 : 27 (dry weight) [10, 11].

Previous studies demonstrated that NXT protected H9c2 cardiomyocytes from H_2_O_2_-induced oxidative injury by enhancing antioxidant abilities, activating ERK1/2 signaling, inhibiting apoptosis-related signal transduction pathways, reducing intracellular Ca^2+^ concentrations, and improving mitochondrial membrane potential [10]. NXT can increase the density of survived pyramidal cells in CA1 region and protect neurons against ischemic stroke in rats [12]. Also, NXT can protect proatherogenic mice against the development of atherosclerosis by ameliorating serum lipid profiles and inhibiting maturation of dendritic cells [13]. NXT has shown the neuroprotective effects in cerebrovascular diseases. However, the neuroprotection mechanisms of NXT on ischemia/reperfusion injured neurons have not yet been well known.

In this study, we will establish the oxygen-glucose deprivation/reoxygenation (OGD/R) induced neurons injury model and treat the neurons with cerebrospinal fluid containing NXT (BNC) to investigate the effects of NXT on OGD/R induced neurons injury and potential mechanisms.

## 2. Materials and Methods

### 2.1. Materials

NXT was purchased from Shanxi Buchang Pharmaceutical Co., Ltd. (Xi'an, China) (drug approval number Z20025001; product batch number 1411145). B27, DCFH-DA, Calcein-AM, and Trizol were obtained from Invitrogen (Eugene, USA). Cell counting kit-8 and Rhod 2-AM probe were obtained from Dojindo (Kumamoto, Japan). DAF-FM DA probe and JC-1 assay kit were from Beyotime (Shanghai, China). Cytochrome-C ELISA kit and neuronal Nitric Oxide Synthase ELISA kit were obtained from Cusabio (Wuhan, China). RIPA buffer, BCA Bradford protein assay kit, poly-D-lysine, and trypsin were purchased from Solarbio (Beijing, China). TaqMan reverse transcription reagents were obtained from Applied Biosystems (Foster City, USA). Cleaved caspase-3 and p-Akt antibodies were obtained from Cell Signaling Technology (Danvers, MA, USA). Bcl-2, Bax, *β*-actin, and HRP-conjugated secondary antibodies were purchased from Abcam (Cambridge, MA, USA).

### 2.2. Preparation of Cerebrospinal Fluid Containing NXT (BNC)

Cerebrospinal fluid containing NXT (BNC) was collected as described previously [14, 15]. All animal experimental procedures were in compliance with the Tianjin University of Traditional Chinese Medicine Guide for Care and Use of Laboratory Animals. 20 health rabbits were administrated intragastrically with 0.262 g/kg/day NXT for 3 days; 20 health rabbits were subjected to ultrapure water. Cerebrospinal fluid was collected from foramen magnum of these rabbits with 1 mL injector. The BNC or blank cerebrospinal fluid (CSF) collected from different rabbits were mixed and centrifuged at 3000 rpm/min for 15 mins and then stored at −80°C.

### 2.3. Isolation and Culture of Primary Cortical Neurons

Primary neurons were prepared from the cortex of embryonic day 16 Wistar rats according to the previous methods [16]. In Brief, cortices were dissected free of meninges and minced and then digested in 0.25% trypsin in Ca^2+^/Mg^2+^ free PBS for 5 minutes at 37°C. The homogenized tissue was forced through a 74 *μ*m nylon sieve. After centrifugation, the pellets were suspended in DMEM/F12 supplemented with 10% FBS, 100 U/mL penicillin, 100 U/L streptomycin, and 2% B-27 and plated onto poly-D-lysine coated 35 mm dish or tissue culture plates. At 4 hours after primary culture, the medium was replaced with serum-free DMEM/F12 supplemented with 100 U/mL penicillin, 100 U/L streptomycin, and 2% B-27. Half of the medium was replaced every 2 days.

### 2.4. Establishment of Oxygen-Glucose Deprivation/Reoxygenation (OGD/R) Model

OGD/R model was established at 7 days of primary neurons culture according to the methods previously described with modifications [17]. OGD/R experiments were performed using a specialized hypoxia incubator chamber (Stem Cell, Canada) that contained an anaerobic gas mixture (95% N_2_ and 5% CO_2_) kept at 37°C. To initiate OGD/R, culture medium was replaced with glucose-free Dulbecco's Modified Eagle's Medium (DMEM) with 10% blank cerebrospinal fluid (volume percent), different-volume (2.5%, 5%, and 10%) BNC plus different-volume (7.5%, 5%, and 0, resp.) blank cerebrospinal fluid (DMEM containing total 10% cerebrospinal fluid), or 10% BNC plus LY294002. After 4 h challenge, cultures were removed from the hypoxia incubator, and the OGD medium in the cultures was replaced with DMEM containing 4.5 mM glucose (pH 7.4). Neurons were then allowed to recover for 2 h in a regular CO_2_ incubator.

### 2.5. Cell Viability and Neurotoxicity

Cell viability was measured using the commercial cell counting kit-8 according to the manufacturer's instructions. Lactate dehydrogenase (LDH) release is an indicator of plasma membrane damage and a commercial LDH assay kit was used for the measurement of neurotoxicity according to the manufacturer's instructions.

### 2.6. Apoptotic Assays

Neurons were harvested with 0.25% trypsin and washed twice with cold D-hank's and then resuspended in 0.1 mL binding buffer containing 5 *μ*L Annexin V-FITC and 5 *μ*L propidium iodide and incubated for 15 min at room temperature. Cellular fluorescence was analyzed with a flow cytometer (BD, USA) with an excitation wavelength of 458 nm and an emission wavelength of 560 nm. Annexin V-FITC positive cells indicated apoptotic neurons, total cell numbers were counted by flow cytometer, and apoptotic rate was calculated by Annexin V-FITC positive cell numbers/total cell numbers ×  %.

### 2.7. Determination of Ca^2+^ Concentration on Cytoplasm and Mitochondria

Ca^2+^ concentration on cytoplasm and mitochondria was determined with Rhod 2-AM probe as described previously [18]. To monitor Ca^2+^ concentration on cytoplasm, neurons were loaded with 4 *μ*M Rhod 2-AM containing 0.05% Pluronic-F127 for 40 minutes at 37°C and then washed with D-hank's three times. The cellular fluorescence was measured at ex/em wavelength of 557/581 nm using a fluorescence microplate reader (Perkin Elmer, USA). Dihydro-Rhod 2-AM enhances the selectivity for mitochondrial loading because this dye exhibits Ca^2+^ dependent fluorescence only after it is oxidized and this occurs preferentially within the mitochondria. To monitor Ca^2+^ concentration in mitochondria, neurons were loaded with 4 *μ*M dihydro-Rhod 2-AM containing 0.05% Pluronic-F127 for 40 minutes at 37°C and then washed with D-hank's three times. The cellular fluorescence was measured at ex/em wavelength of 553/576 nm using a fluorescence microplate reader.

### 2.8. Measurement of Intracellular Reactive Oxygen Species (ROS) and NO Levels

The intracellular ROS production was measured as described previously [19]. Neurons were loaded with 5 *μ*M DCFH-DA at 37°C for 30 minutes and then washed with D-hank's three times to remove residual probe. The cellular fluorescence was measured with a fluorescence microplate reader at an ex/em wavelength of 488/525 nm. Results were expressed as a percentage of the control group. DAF-FM DA probe was used to detect intracellular NO levels [20]. Neurons were loaded with 5 *μ*M DAF-FM DA at 37°C for 20 minutes and then washed with PBS three times to remove residual probe. The cellular fluorescence was measured with a fluorescence microplate reader at an ex/em wavelength of 495/515 nm. Results were expressed as a percentage of the control group.

### 2.9. Measurement of Mitochondrial Membrane Potential (ΔΨ*m*)

JC-1 assay kit was used to measure the mitochondrial membrane potential [21]. Neurons were loaded with JC-1 for 20 min at 37°C and then washed with cold JC-1 staining buffer twice. JC-1 could induce J-aggregates when ΔΨ*m* levels were high, J-aggregates produced red fluorescence at an ex/em wavelength of 525/590 nm, JC-1 produced green fluorescence at an ex/em wavelength of 490/530 nm, and the ratio of fluorescence red to green was expressed as the ΔΨ*m* levels in neurons.

### 2.10. Measurement of Mitochondrial Permeability Transition Pore (mPTP) Opening

mPTP opening was measured by analyzing the calcein leaks as described previously [22]. Calcein-AM is permeable to the mitochondria. Calcein leaks from the mitochondria when mPTP opens; Co^2+^ can quench the calcein fluorescence, so we can detect the level of mPTP opening by this method. To measure mPTP opening, neurons were loaded with Calcein-AM (2 *μ*M) and CoCl_2_ (1 *μ*M) for 40 min at dark. Fluorescence was detected with emission wavelength at 515 nm and excitation wavelength at 488 nm using a fluorescence microplate reader.

### 2.11. Measurement of the nNOS and Cytochrome-c

Culture medium was collected at 2 hours after reoxygenation; nNOS and cytochrome-c were measured using rat neuronal Nitric Oxide Synthase ELISA Kit and rat Cytochrome-c ELISA Kit according to the manufacturer's instructions, respectively.

### 2.12. Measurement of Caspase-3 Activity

Caspase-3 activity was measured using the Live Cell Caspase-3/7 and Phosphatidylserine Detection Kit according to the manufacturer's instructions. SR-DEVD-FMK was the fluorescence indicator of caspase-3/7 activity and detected with emission wavelength at 595 nm and excitation wavelength at 550 nm using a fluorescence microplate reader.

### 2.13. Quantitative Real-Time PCR Analysis

Total RNA from neurons was extracted using Trizol and was reverse transcribed by TaqMan reverse transcription reagents. All primers were provided by Sangon Biotech (Shanghai, China). The Rat ACTB Endogenous Reference Genes Primers (Cat. PRN02) could obtain more accurate target gene expression (Sangon Biotech, Shanghai, China); the sense and antisense primers for Bcl-2, Bax, caspase-3, caspase-9, and nNOS are described in Table 1. The amplification was accomplished in an ABI 7500 Real-Time PCR System (Applied Biosystems, Foster City, USA). The expression of related target genes was determined by the 2-ΔΔCT method.

### 2.14. Western Blot Analysis

After treatment, neurons were washed with ice cold D-hank's and lysed in RIPA buffer. Protein concentration was measured using the BCA Bradford protein assay kit and mixed with SDS-PAGE loading buffer. Following heating at 100°C for 8 min, proteins were subjected to 10–12% SDS-PAGE gel and transferred electrophoretically to polyvinylidene difluoride membranes (Millipore, USA) and then blocked with 5% nonfat milk in PBS-T (PBS containing 0.1% Triton X-100) for 2 hours at room temperature. Incubation with primary antibody against Bcl-2 (1 : 1000), Bax (1 : 1000), cleaved caspase-3 (1 : 1000), p-Akt (1 : 1000), and *β*-actin (1 : 1000) was done overnight at 4°C and then with HRP-conjugated secondary antibody (1 : 2000). Antibody positive bands were detected with an enhanced chemiluminescence agent (Millipore, USA). The band densities were quantified by Image J software.

### 2.15. Statistical Analysis

Data were expressed as mean ± SD. One-way ANOVA was used to determine significant differences among all groups. *P* values less than 0.05 were considered to be significant. Tamhane's h2 was used for heterogeneity of variance.

## 3. Results

### 3.1. BNC Protected Neurons Injured by OGD/R

**Figure 1:**
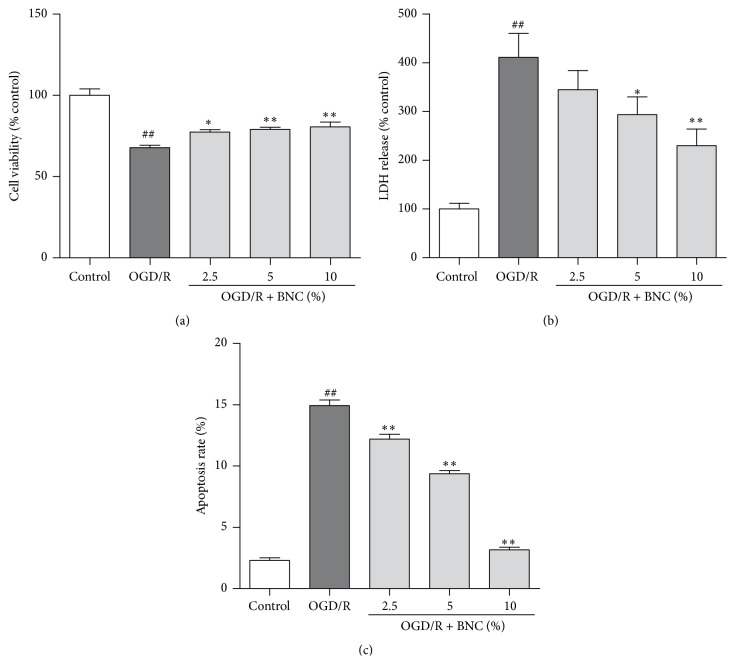
Neuroprotective effects of BNC on OGD/R induced injury in neurons. (a) Cell viability was measured by CCK-8 assay. (b) LDH release. (c) Apoptosis analysis was detected by flow cytometry. Data are expressed as mean + SD. *n* = 6 per group. ^##^ *P* < 0.05 compared with control; ^*∗*^ *P* < 0.05 compared with OGD/R; and ^*∗∗*^ *P* < 0.01 compared with OGD/R.

OGD/R induced a 67.93 ± 3.51% reduction in cell viability; treatment with 2.5%, 5%, and 10% BNC improved cell viability compared with OGD/R group (Figure 1(a)). LDH release was increased following OGD/R, while 2.5%, 5%, and 10% BNC suppressed LDH release from neurons to culture medium compared with OGD/R group (Figure 1(b)). OGD/R significantly increased the apoptosis rates of neurons to 14.93 ± 0.80% at 2 h after reoxygenation; 2.5%, 5%, and 10% BNC decrease in a 18.3%, 37.2%, and 78.8% reduction in apoptosis rates were detected by flow cytometry compared with OGD/R group, respectively (Figure 1(c)).

### 3.2. Effects of BNC on Cytosolic and Mitochondrial Ca^2+^ in Neurons Injured by OGD/R

**Figure 2:**
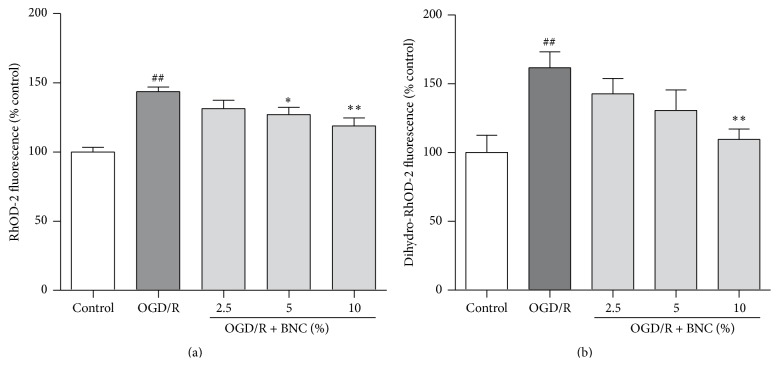
Effects of BNC on calcium overload in neurons following OGD/R induced injury. (a) Ca^2+^ concentration in cytoplasm indicated by Rhod 2 fluorescence. (b) Ca^2+^ concentration in mitochondria indicated by dihydro-Rhod 2 fluorescence. Data are expressed as mean + SD. *n* = 6 per group. ^##^ *P* < 0.01 compared with control; ^*∗*^ *P* < 0.05, ^*∗∗*^ *P* < 0.01 compared with OGD/R.

OGD/R induced a significant increase in cytosolic and mitochondrial Ca^2+^ levels to 143.62 ± 8.18% and 161.57 ± 28.37% compared with control group, respectively. Treatment with 2.5%, 5%, and 10% BNC decreased Ca^2+^ levels on cytoplasm and mitochondria compared to OGD/R group; particularly 10% BNC treatment significantly decreased both cytosolic and mitochondrial Ca^2+^ levels (Figures 2(a) and 2(b)).

### 3.3. BNC Decreased Intracellular ROS and NO following OGD/R Injury

**Figure 3:**
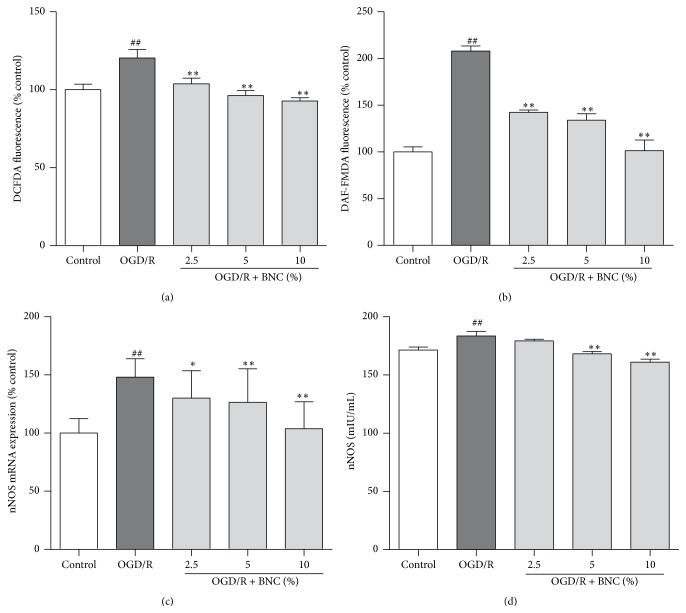
Effects of BNC on ROS generation, NO and nNOS expression in neurons following OGD/R induced injury. (a) The intracellular ROS generation was indicated by DCFDA fluorescence. (b) The intracellular NO production was indicated by DAF-FM DA fluorescence. (c) nNOS mRNA expression. (d) nNOS levels in neuron culture medium. Data are expressed as mean + SD. *n* = 6 per group. ^##^ *P* < 0.01 compared with control; ^*∗*^ *P* < 0.05, ^*∗∗*^ *P* < 0.01 compared with OGD/R.

OGD/R induced a significant increase in intracellular ROS generation and NO levels to 120.35 ± 13.11% and 208.09 ± 13.16% compared with control group, respectively. Treatment with 2.5%, 5%, and 10% BNC significantly decreased intracellular ROS generation and NO levels compared with OGD/R group (Figures 3(a) and 3(b)). OGD/R increased intracellular nNOS mRNA expression to 209 ± 67% and nNOS levels of culture medium to 183.43 ± 9.64 mIU/mL compared with control group; 2.5%, 5%, and 10% BNC significantly decreased intracellular nNOS mRNA levels to 136 ± 41%, 113 ± 0.47%, and 97 ± 29% compared to OGD/R group, respectively (Figures 3(c) and 3(d)). Treatment with 5% and 10% BNC reduced nNOS concentration of culture medium to 179.23 ± 3.76 mIU/mL and 160.96 ± 6.31 mIU/mL (Figure 3(d)).

### 3.4. Effects of BNC on mPTP Opening, Cytochrome-c Release, and ΔΨ*m* in OGD/R Injured Neurons

**Figure 4:**
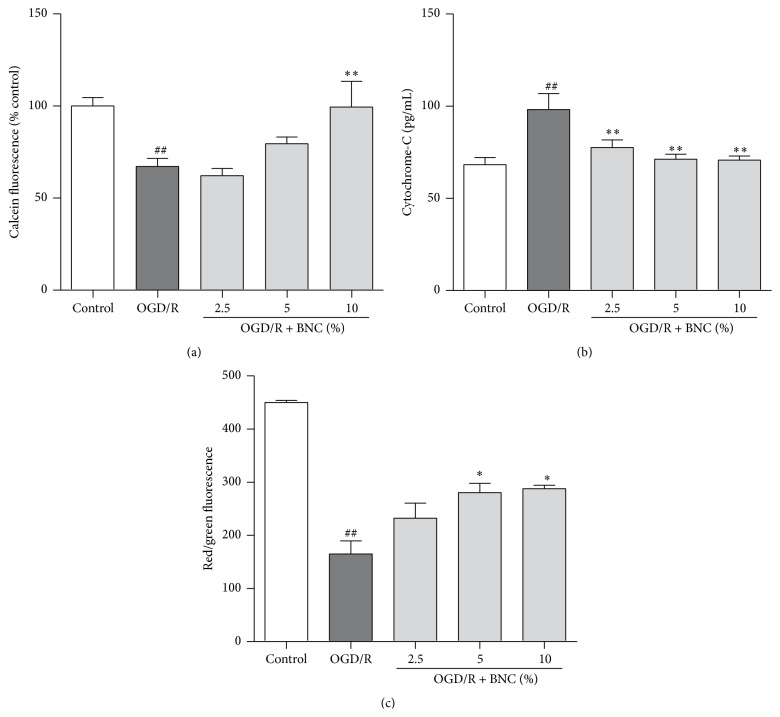
Effects of BNC on mPTP opening, cytochrome-c release, and ΔΨ*m* in neurons following OGD induced injury. (a) mPTP opening was detected by calcein fluorescence. (b) Cytochrome-c release was measured by ELISA. (c) ΔΨ*m* was indicated by the ratio of fluorescence red (J-aggregates) to green (JC-1). Data are expressed as mean + SD. *n* = 6 per group. ^##^ *P* < 0.01 compared with control; ^*∗*^ *P* < 0.05, ^*∗∗*^ *P* < 0.01 compared with OGD/R.

OGD/R resulted in a significant decrease in calcein fluorescence to 67.20 ± 10.76% compared with control group, indicating increased mPTP opening; 10% BNC treatment attenuated OGD/R induced mPTP opening (Figure 4(a)). OGD/R induced the release of cytochrome-c, while 2.5%, 5%, and 10% BNC treatment inhibited the release of cytochrome-c after OGD/R (Figure 4(b)). OGD/R reduced ΔΨ*m* to 36.65 ± 13.55% compared to control group, while treatment with 5% and 10% BNC attenuated the reduction in ΔΨ*m* when compared with OGD/R group (Figure 4(c)).

### 3.5. Effects of BNC on Bcl-2, Bax, Caspase-3, and Caspase-9 Expression in OGD/R Injured Neurons

**Figure 5:**
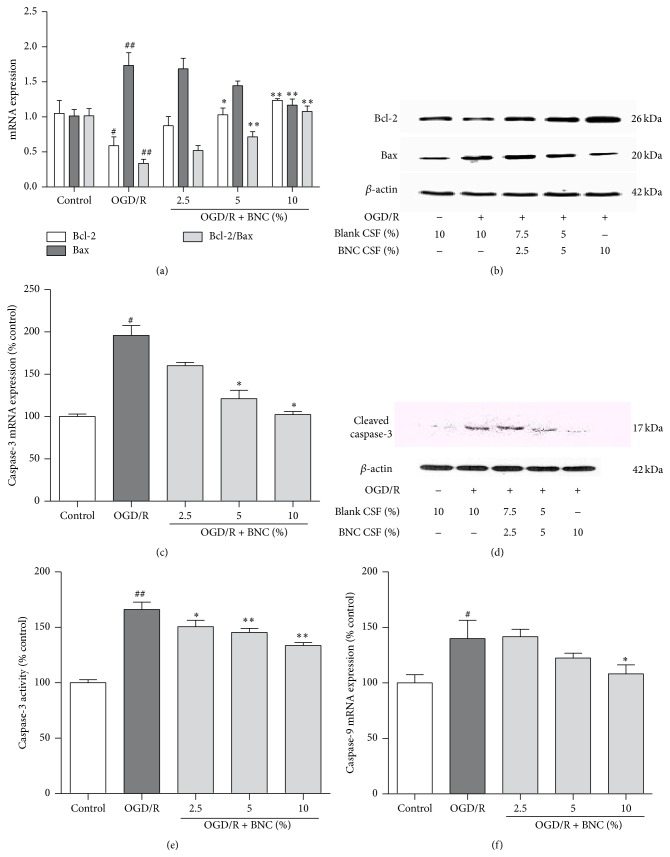
Effects of BNC on apoptosis related proteins expression in neurons following OGD/R induced injury. (a) Bcl-2 and Bax mRNA expression detected by real-time RT-PCR. (b) Representative image of Bcl-2 and Bax expression detected by Western blot. (c) Caspase-3 mRNA expression. (d) Representative image of cleaved caspase-3 expression detected by Western blot. (e) Caspase-3 activity. (f) Caspase-9 mRNA expression. Data are expressed as mean + SD. *n* = 6 per group. ^#^ *P* < 0.05, ^##^ *P* < 0.01 compared with control; ^*∗*^ *P* < 0.05, ^*∗∗*^ *P* < 0.01 compared with OGD/R.

OGD/R strongly decreased Bcl-2 mRNA expression, increased Bax mRNA expression, and diminished the ratio of Bcl-2/Bax mRNA expression compared with control group, while 5% and 10% BNC treatment significantly increased Bcl-2 mRNA expression, and treatment with 10% BNC decreased Bax mRNA expression; meanwhile treatment with 5% and 10% BNC upregulated the ratio of Bcl-2/Bax (Figure 5(a)). Similar results were confirmed on the protein expression levels: OGD/R decreased Bcl-2 expression and increased Bax expression compared with control group; treatment with 2.5%, 5%, and 10% BNC increased Bcl-2 expression and decreased Bax expression (Figure 5(b)).

OGD/R significantly increased caspase-3 and caspase-9 mRNA expression, and treatment with BNC downregulated capase-3 mRNA and caspase-9 mRNA expression compared with OGD/R group (Figures 5(c) and 5(f)). Meanwhile, 2.5%, 5%, and 10% BNC treatment decreased cleaved caspase-3 expression and caspase-3 activity (Figures 5(d) and 5(e)).

### 3.6. BNC Increased the Phosphorylation of Akt following OGD/R Injury

**Figure 6:**
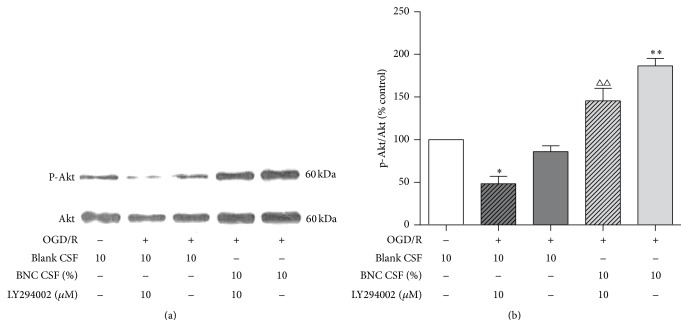
Effects of BNC on p-Akt expression in neurons following OGD/R induced injury. (a) Representative image of p-Akt expression detected by Western blot. (b) Quantitative analysis of p-Akt expression. Data are expressed as mean + SD. *n* = 6 per group. ^*∗*^ *P* < 0.05, ^*∗∗*^ *P* < 0.01, compared with OGD/R. ^ΔΔ^ *P* < 0.01, compared with OGD/R + BNC.

OGD/R decreased the levels of phosphorylated Akt compared with control group; treatment with 10% BNC increased phosphorylation of Akt following OGD/R. LY294002, the inhibitor of PI3 kinase, blocks Akt activation both in vivo and in vitro [23] and significantly partially attenuated the 10% BNC induced increase of phosphorylation of Akt (Figures 6(a) and 6(b)).

### 3.7. Effects of LY2940002 on Ca^2+^ Overload, ROS Generation, mPTP Opening, and Caspase-3 Activity in OGD/R Injured Neurons

**Figure 7:**
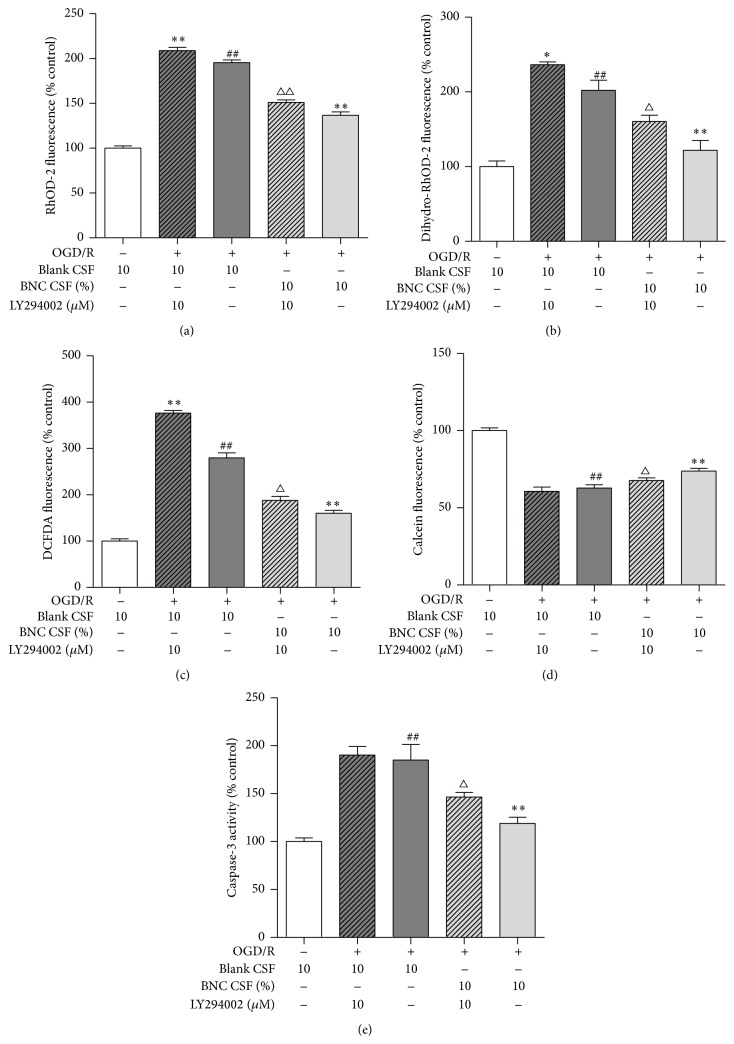
Effects of LY2940002 on Ca^2+^ overload, ROS generation, mPTP opening, and caspase-3 activity in OGD/R injured neurons. (a) Effect of LY2940002 on Ca^2+^ concentration in cytoplasm. (b) Effect of LY2940002 on Ca^2+^ concentration in mitochondria. (c) Intracellular ROS generation was indicated by DCFDA fluorescence. (d) mPTP opening was detected by calcein fluorescence. (e) Caspase-3 activity. Data are expressed as mean + SD. *n* = 6 per group. ^##^ *P* < 0.01, compared with control. ^*∗*^ *P* < 0.05, ^*∗∗*^ *P* < 0.01, compared with OGD/R. ^Δ^ *P* < 0.05, ^ΔΔ^ *P* < 0.01, compared with OGD/R + BNC.

LY294002 significantly partially attenuated 10% BNC induced reduction of cytosolic and mitochondrial Ca^2+^ levels compared with BNC treatment alone (Figures 7(a) and 7(b)). LY294002 significantly partially obscured 10% BNC induced reduction of intracellular ROS levels compared with BNC treatment alone (Figure 7(c)). LY294002 slightly depressed 10% BNC induced inhibition of mPTP opening (Figure 7(d)). LY294002 significantly partially attenuated 10% BNC induced reduction of caspase-3 activity compared with BNC treatment alone (Figure 7(e)).

## 4. Discussion

NXT is a traditional Chinese medicine approved by the Chinese National Drug Administration and used to treat patients with stroke and cardiovascular diseases, which contains 16 various kinds of traditional Chinese medicines. Because of blood brain barrier and the specific survival environment of neurons in central nervous system, drug-containing serum, intestinal absorption liquid, and extraction solution have some limitations to study the effects of traditional Chinese medicines for protecting neurons in vitro. Cerebrospinal fluid containing NXT (BNC) was used in this study instead of drug-containing serum, intestinal absorption liquid, and extraction solution. Cerebrospinal fluid pharmacology is an emerging and improved pharmacological method which using cerebrospinal fluid was extracted from animals after administration of traditional Chinese medicines to explore the material basis and the efficacy mechanisms for treating neural cells including neuron and glial cells in vitro [14, 15].

BNC showed the protective effects against OGD/R induced neurons injury by improving cell viability and decreasing LDH release. As previously described, apoptosis is an important form and stage of neuron death during OGD/R injury [24]. In the present study, BNC reduced the numbers of apoptotic cells after OGD/R injury as detected by flow cytometry following Annexin-V staining. Mammalian cell apoptosis induced by ischemia-reperfusion is strongly dependent on the mitochondria [25]. The mPTP is a Ca^2+^-, ROS-dependent high-conductance channel and appears to be critical for the cerebral ischemia-reperfusion injury; numerous neuroprotective mechanisms are associated with the inhibition of mPTP opening [26]. Previous studies suggested that ROS could increase mPTP opening during ischemia-reperfusion [27]. Transient opening of mPTP is linked to the release of cytochrome-c, which activates downstream apoptotic cascades [28]. As shown in Figure 4(a), BNC depressed OGD/R induced mPTP opening. The release of cytochrome-c from mitochondria to the cytoplasm is a crucial process in cell apoptosis [24]; BNC reduced the release of cytochrome-c from mitochondria following OGD/R (Figure 4(c)).

Previous studies showed that a rise in intramitochondrial Ca^2+^ increases ROS production [29], the combination triggers opening of the mPTP, and this leads to ATP depletion and the cells' death [30]. To monitor Ca^2+^ concentration in cytoplasm and mitochondria, Rhod 2-AM and dihydro-Rhod 2-AM were used, respectively. OGD/R markedly increased Rhod 2-AM and dihydro-Rhod 2-AM fluorescence, indicating calcium overload in the cytoplasm and mitochondria. BNC significantly suppressed both cytosolic and mitochondrial Ca^2+^ overload. ΔΨ*m* is important for normal neuronal activity and mitochondrial function and contributes to ATP production, intracellular Ca^2+^ signaling, and generation of ROS [31]. Loss of ΔΨ*m* is the earliest event that commits the neuron to death. There were a progressive ΔΨ*m* loss during the ischemic period and a more prominent ΔΨ*m* loss on reperfusion [32]. Our results showed that BNC attenuated the reduction in ΔΨ*m* compared with OGD/R group.

The Bcl-2 and Bax are crucial checkpoints that control apoptosis in the mitochondria [33]. Bax-mediated cytochrome-c release occurs by a selective increase in the permeability of mitochondria [34]. Pro-caspase-3 is downstream of cytochrome-c in the apoptotic cascades and cleaved by active caspase-9 to produce active caspase-3. Cytochrome-c released from mitochondria combines with pro-caspase-9 in the cytosol to produce active caspase-9, which cleaves procaspase-3 to activate caspase-3 and initiates apoptosis [33, 35]. In the present study, OGD/R strongly diminished the ratio of Bcl-2/Bax mRNA expression and increased caspase-3 mRNA expression and activity compared with control group, while BNC treatment significantly upregulated the Bcl-2/Bax ratio, downregulated capase-3 mRNA expression, and increased caspase-3 activity.

PI3K-Akt pathway has been demonstrated to be crucial in modulating brain cell death and survival after ischemic cascades in both in vivo and in vitro studies [36, 37]. Phosphorylated Akt may protect neurons from cell death by inducing BAD phosphorylation, which will not bind with Bcl-2 family protein, free Bcl-2 has the antiapoptotic effects [38], and Akt promotes cell survival by intervening in the apoptosis cascades before cytochrome-c release and caspase activation via a mechanism that is distinct from BAD phosphorylation [39]. Akt inhibits activation of caspase-9 and caspase-3 by posttranslational modification of a cytosolic factor downstream of cytochrome-c and before activation of caspase-9 [40]. In the present study, BNC increased phosphorylation of Akt following OGD/R. Moreover, LY294002 further inhibited phosphorylation of Akt following OGD/R and partially attenuated the BNC induced increase of phosphorylated Akt.

In the current study, we used cerebrospinal fluid containing NXT (BNC) to explore the effects of NXT on neurons injured by OGD/R and demonstrated the neuroprotective effects and mechanisms of NXT. As we know, the NXT contains 16 various kinds of traditional Chinese medicines, and the components are very complex; each traditional Chinese medicine may contain hundreds of chemical constituents [41]. Further experiments to determine the main components of the cerebrospinal fluid containing NXT and confirm which components could transport across blood brain barrier would be clinically important.

## 5. Conclusions

Our study demonstrated that NXT protected primary neurons from OGD/R induced injury by inhibiting calcium overload and ROS generation, protecting mitochondria, and inhibiting mitochondrial apoptotic pathway which was mediated partially by PI3K-Akt signaling pathway activation.

## Figures and Tables

**Table 1:** The sense and antisense primers.

Genes	Primer/probe	Primer/probe sequences (5′ to 3′)
Bcl-2	Forward primer	5′-GAGCGTCAACAGGGAGATGT-3′
	Reverse primer	5′-CAGCCAGGAGAAATCAAACAG-3′
Bax	Forward primer	5′-TTGCTACAGGGTTTCATCCA-3′
	Reverse primer	5′-TGTTGTTGTCCAGTTCATCG-3′
Caspase-3	Forward primer	5′-AGCTGGACTGCGGTATTGAG-3′
	Reverse primer	5′-GGGTGCGGTAGAGTAAGCAT-3′
Caspase-9	Forward primer	5′-TATGGCACAGATGGATGCTC-3′
	Reverse primer	5′-CTTTCTGCTCACCACCACAG-3′
nNOS	Forward primer	5′-TGGCAGCCCTAAGACCTATG-3′
	Reverse primer	5′-AGTCCGAAAATGTCCTCGTG-3′
